# Proven infection-related sepsis induces a differential stress response early after ICU admission

**DOI:** 10.1186/cc9102

**Published:** 2010-07-09

**Authors:** Olivier Lesur, Jean-Francois Roussy, Frederic Chagnon, Nicole Gallo-Payet, Robert Dumaine, Philippe Sarret, Ahmed Chraibi, Lucie Chouinard, Bruno Hogue

**Affiliations:** 1 Soins intensifs médicaux, département de Médecine, Université de Sherbrooke, 3001, 12th Avenue Nord, Sherbrooke, QC J1H 5N4, Canada; 2 Centre de recherche clinique Étienne-Lebel (CRCEL) CHUS, Université de Sherbrooke, 3001, 12th Avenue Nord, Sherbrooke, QC J1H 5N4, Canada; 3 Département de Physiologie et Biophysique, Université de Sherbrooke, 3001, 12th Avenue Nord, Sherbrooke, QC J1H 5N4, Canada

## Abstract

**Introduction:**

Neuropeptides arginine-vasopressin (AVP), apelin (APL), and stromal-derived factor-1α (SDF-1α) are involved in the dysfunction of the corticotropic axis observed in septic ICU patients. Study aims were: *(i) *to portray a distinctive stress-related neuro-corticotropic systemic profile of early sepsis, *(ii) *to propose a combination data score, for aiding ICU physicians in diagnosing sepsis on admission.

**Methods:**

This prospective one-center observational study was carried out in a medical intensive care unit (MICU), tertiary teaching hospital. Seventy-four out of 112 critically ill patients exhibiting systemic inflammatory response syndrome (SIRS) were divided into two groups: proven sepsis and *non sepsis*, based on *post hoc *analysis of microbiological criteria and final diagnosis, and compared to healthy volunteers (n = 14). A single blood sampling was performed on admission for measurements of AVP, copeptin, APL, SDF-1α, adrenocorticotropic hormone (ACTH), cortisol baseline and post-stimulation, and procalcitonin (PCT).

**Results:**

Blood baseline ACTH/cortisol ratio was lower and copeptin higher in septic vs. nonseptic patients. SDF-1α was further increased in septic patients vs. normal patients. Cortisol baseline, ACTH, PCT, APACHE II and sepsis scores, and shock on admission, were independent predictors of sepsis diagnosis upon admission. Using the three first aforementioned categorical bio-parameters, a probability score for predicting sepsis yielded an area under the Receiver Operating Curve (ROC) curves better than sepsis score or PCT alone (0.903 vs 0.727 and 0.726: *P *= 0.005 and *P *< 0.04, respectively).

**Conclusions:**

The stress response of early admitted ICU patients is different in septic vs. non-septic conditions. A proposed combination of variable score analyses will tentatively help in refining bedside diagnostic tools to efficiently diagnose sepsis after further validation.

## Introduction

Severe sepsis and septic shock are the most common causes of death in intensive care units (ICU) with a mortality rate ranging from 30 to 70% [1]. Most of these related deaths result from multiple organ dysfunction/failure occurring in advanced stages of septic shock [2]. During septic shock, the hypothalamo-pituitary-adrenal (HPA) axis is also dysfunctional and the neuroendocrine stress response system is disrupted [2,3]. One of the best examples is the concept of relative adrenal insufficiency which has recently been denominated CIRCI (critical illness-related corticosteroid insufficiency) [4]. Annane *et al*. first demonstrated that septic shock patients exhibiting a blunted dynamic cortisol response to corticotropin had more vasopressor-unresponsive shock and a higher 28-day mortality rate [5,6]. The Corticosteroid therapy of septic shock (CORTICUS) study confirmed almost half of septic patients exhibited an inappropriate dynamic response to corticotropin, and that septic shock was more quickly reversed for those receiving hydrocortisone [7].

Neuropeptides of the posterior pituitary area may be actively committed in the regulation of the aforementioned corticotropic axis. Indeed, arginine-vasopressin (AVP) heightens hypothalamic sensitivity to corticotrophin-releasing hormone (CRH), thereby increasing ACTH release and cortisol production, and improves hemodynamics during sepsis [8,9]. Apelin (APL), a new endogenously released peptide discovered in 1998 and co-expressed within vasopressinergic neurons, was found to be implicated in the up-regulation of ACTH pituitary secretion [9-11]. Furthermore, the stromal-derived factor-1α (SDF-1α), a member of the chemokine family recently reported to co-localize with AVP in the magnocellular neurons of the hypothalamic supraoptic (SON) and paraventricular nuclei (PVN), may potentially interfere with AVP functions [12].

It is unknown whether the stress response to ICU admission leads to a uniform pathway, irrespective if the cause of admission is sepsis-related or not, whether neuropeptide release can interact with the neighboring corticotrope axis in stress conditions, and if the outcome can be affected by their ensuing cross-talks. In parallel, the search for biomarkers to improve diagnostic accuracy in systemic infections is especially relevant in an ICU setting. Recent reports have emphasized the value of measuring blood procalcitonin (PCT), C-reactive protein in the initial diagnostic assessment of systemic infection [13]. Copeptin, a stable moiety of pre-pro-AVP is also considered as a valuable marker of severe sepsis [14-16]. However, there is still room for improvement in establishing a robust protocol, especially with regards to the imperfect predictive value of PCT for sepsis-specific diagnosis.

The main hypothesis is that a differential molecular profile can be observed depending on the stressor's origin, with correlations evoking cross-regulation, and that the related initial blood profile can predict the diagnosis of sepsis. A primary objective of the present study was to outline a profile of neuro-corticotropic systemic blood content in two stress causative groups of early admitted MICU patients and to tentatively delineate differential response patterns. A secondary objective was to determine, in a *post hoc *analysis, the best interpretative risk assessment score, including an overview of neuro-corticotropic molecules, which could further support conventional microbiological samples to help ICU physicians in the early diagnosis of sepsis upon MICU admission.

## Materials and methods

### Patients

This study was conducted between December 2007 and 2008 in a 16-bed medical and coronary intensive care unit (MICU) of a tertiary university teaching hospital admitting 1,000 to 1,500 patients/year.

**Figure 1:**
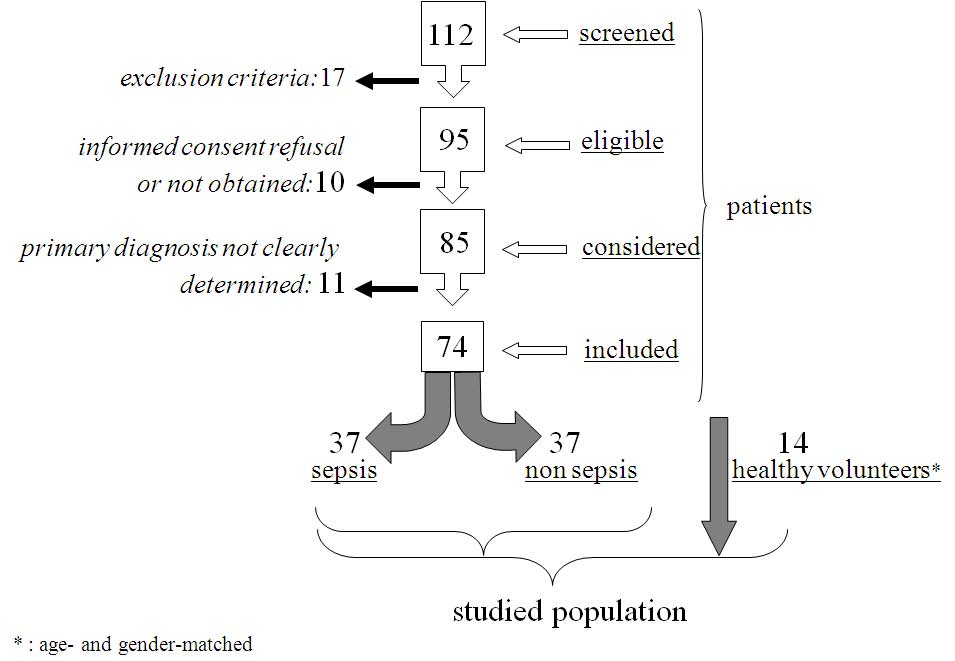
**Study design**. Grouping process of studied patients and volunteers.

Seventy-four out of the 112 screened patients exhibiting systemic inflammatory response syndrome (SIRS) were consecutively included within the first 24 hours of admission to MICU (Figure 1). From this cohort, two groups of patients were compared to one control group: A first group (so called *sepsis*) of patients demonstrating severe sepsis or septic shock according to criteria of the 2001 SCCM/ESICM/ACCP/ATS/SIS International Sepsis Definitions Conference and surviving sepsis campaign guidelines [17,18], further proven by microbiological evidence of infection; a second group (so called *non-sepsis*, as an internal control stressed group) comprised of patients admitted for critical illness with a clearly documented noninfectious primary cause and without evidence of infection; a third group of healthy gender- and age-matched non-hospitalized volunteers was subsequently recruited.

The APACHE II score, and in-hospital mortality were also recorded for MICU patients. In addition, a sepsis score was *post hoc *calculated for the sepsis group, taking into account: general; inflammatory (including PCT); hemodynamic; organ dysfunction; and tissue perfusion variables (maximum 19 points, scheme 1 in [18]).

Group allocation, with respect to microbiological data; and final primary diagnosis, was performed blindly to biomarkers' results and sepsis score calculation and *post hoc *validated after in-hospital file examination by three independent researchers. Based on the primary reason of admission; five re-allocations per group (n = 10, 13.5%) were processed after file analysis.

Patients were excluded if under 18 years old, pregnant, having contra-indications to receiving corticotropin, treated in the past three months with corticosteroids, requiring administration of corticosteroids; drug(s) associated with corticosteroid insufficiency (for example, etomidate); or exogenous AVP infusion in the present admission. Patients in septic shock requiring corticosteroids were not excluded because they were sampled before the treatment initiation.

This study was approved by the institutional review board and the ethics committee of the Sherbrooke University Medical Center. Consents were obtained directly from patients or next of kin after permission from the on-duty ICU physician and specific approval for adrenal gland sampling of organ donors.

### Blood collection in critically ill patients

Blood samplings were always performed between 9 and 11 AM. A first sampling was collected in iced aprotinin (0.33 U, Sigma-Aldrich, St. Louis, MO, USA) -containing tubes, and plasma preserved for AVP, copeptin, APL, ACTH and SDF-1α CXCL12 measurements. Remaining blood was used for establishing cortisol baseline after which 250 μg of cosyntropin/corticotropin (Cortrosyn^®^, Amphastar Pharmaceuticals, Scarborough, ONT, Canada) was injected intravenously, and blood was retrieved 30 minutes and 60 minutes later. Serum lactate and albumin values were also quantified on the first day of admission, as described previously [19].

### Determination of blood cortisol and ACTH concentrations

Blood cortisol and ACTH measurements were performed as detailed in Table 1. The Δmax cortisol after corticotropin challenge was determined as a marker of adrenal gland reserve response by subtracting the cortisol baseline value from the 60 minutes cortisol value. CIRCI was defined as Δmax ≤ 248 nMol/L or cortisol baseline ≤ 276 nMol/L, as recommended [4].

**Table 1:** Specifications of neuropeptide/protein blood dosages

Protein/Peptide	Extraction	Assay	Source	Sensitivity	Range
Cortisol	None	ADVIA Centaur system	Siemens Medical Solutions Diagnostics, Tarrytown, NY, USA.	5.5 to 2069 nMol/L	85 to 618 nMol/L
ACTH	None	ImmuChem^TM 125^I RIA kit	MP Biomedicals, LLC, Diagnostics Division, Orangeburg, NY, USA.	5.7 pg/mL	0 to 1000 pg/mL
Apelin	C-18 SEP-column and lyophilisation	Apelin-12 enzyme immunoassay kit	Phoenix Pharmaceuticals Inc, Burlingame, California, USA.	0.07 ng/mL	0 to 100 ng/mL
Arg Vasopressin (AVP)	Acetone and ether procedure	Arg^8^-Vasopressin enzyme Immunoassay kit	Assay Designs, Ann Arbor, MI, USA.	3.39 pg/mL	3.39 to 1,000 pg/mL
Copeptin	C-18 SEP-column and lyophilisation	Copeptin enzyme immunoassay kit	R&D Systems, Minneapolis, Minnesota, USA.	18 pg/mL	0 to 1,000 pg/mL
SDF-1α	Centrifuged for complete platelet removal	SDF-1α enzyme immunoassay	R&D Systems, Minneapolis, Minnesota, USA.	18 pg/mL	0 to 10,000 pg/mL
Procalcitonin (PCT)	None	Immunoluminometric	PCT LIA B.R.A.H.M.S Diag LLC, Georgia, USA	0.3 ng/mL	0.3 to 500 ng/mL

ACTH, adreno corticotropic hormone. AVP, arginine vasopressin. SDF-1α, stromal-derived factor-1 alpha. PCT, procalcitonin.

### Determination of circulating neuropeptide and procalcitonin (PCT) concentrations

APL, copeptin, AVP, SDF-1α and PCT blood contents were measured as detailed in Table 1.

### Immunofluorescence imaging of human adrenal glands

Tissue sections were incubated with polyclonal goat anti-C-X-C chemokine receptor type 4 (CXCR4), the chemokine receptor for CXCL12/SDF-1α (sc-6190, 1:50; Santa Cruz Biotechnology, Santa Cruz, CA, USA), and polyclonal rabbit anti-cytochrome P450 steroid 21-hydroxylase (P450c21), a key-limiting enzyme of the adrenal cortex for 11-deoxycortisol and -deoxycorticosterone production, (1:100; gift of Walter L. Miller, University of California, San Francisco, CA, USA) in 1.5% donkey serum (Zymed, San Francisco, CA, USA). Anti-CXCR4 staining was revealed with FITC-conjugated donkey anti-goat IgG and P450c21 with TRITC-conjugated donkey anti-rabbit IgG (1:50; Santa Cruz Biotechnology for both secondary antibodies) in 2% donkey serum. Sections were analyzed using an Axioskop 2 fluorescence microscope (Carl Zeiss, Inc, Thornwood, NY, USA).

### Statistical analysis

Results are presented as median and interquartile ranges in the illustrations, and as mean ± SD in Table 2 (with exception to lactates). Categorical variables are presented as frequency and percentage. Baseline characteristics of septic and nonseptic patients were compared using the Mann-Whitney U test for quantitative variables. A Kruskal-Wallis test with Dunn's multiple comparisons post-test was used for subset analysis and comparisons with healthy subjects, and a Chi-square test (or the Fisher's exact test when frequency was less than five) was selected for proportion comparisons. Correlations between molecular parameters were analyzed using the Spearman r test. Models were built up sequentially starting with the variable most strongly associated with sepsis diagnosis and continuing until no other variable reached significance. When the final model was reached, each variable was dropped in turn to assess its effect. Different models were compared using the likehood ratio test, keeping in the final one variables significant at the *P *= 0.05 level.

**Table 2:** General characteristics of studied groups*

	Non-septic (n = 37)	Septic (n = 37)	*P-*value
Age	60.2 ± 15.3	59.8 ± 17	*0.9164*
Gender (M/F)	22/15	22/15	*1.000*
Apache II score	18.8 ± 8	21.8 ± 6.9	*0.082*
Sepsis score †	6.5 ± 3	8.8 ± 2.1	*0.0005*
Albumin ‡	32.1 ± 7.4	23.5 ± 5.1	*0.0001*
Shock ††	5	19	*0.001*
Lactates ‡	1.25 (0.94 to 2.5)	1.725 (1 to 3.9)	*0.2113*
Kidney dysfunction §	24	25	*1.0000*
CIRCI **			
baseline **\**	4 **\**	1 **\**	*0.3575*
	19%	37%	
after corticotropin **/**	3 **/**	9 **/**	*0.1123*
survivors/nonsurvivors	3/4	4/6	*1.0000*

CIRCI: critical illness-related corticosteroid insufficiency.

*: data are expressed as means ± SD, number of patients or medians with interquartile ranges.

†: according to the 2001 SCCM/ESICM/ACCP/ATS/SIS International Sepsis

Definitions Conference [19].

††: as defined by the need of (inotropic and/or vasopressive) catecholamines or mechanical device (for example, IABP) after at least 6 hrs of initial resuscitation and in order to maintain mean arterial pressures ≥ 65 mmHg.

‡: Albumin (normal range 35 to 50 g/L), lactates (normal scale: 0.7 to 2.1 mmol/L).

§ according to the RIFLE criteria [21].

**: defined according to [4].

Different univariate logistic regression models were performed to evaluate which biological or clinical parameters can predict early sepsis diagnosis. Variables included in the analyses were: (i) cortisol baseline; ACTH, apelin, SDF-1α, AVP, copeptin, PCT (for molecular parameters), as well as (ii) age; APACHE II score, sepsis score, gender, shock on admission, (continuous variables for the three formers and binary for the two last parameters). Because normal distribution of biological values was not reached, the selected parameters were categorized: cutoffs values were determined by optimal likelihood ratios of individual receiver operating curve (ROC) analysis, or according to the manufacturer's recommendation for PCT.

Different multivariate logistic regression models with a stepwise selection procedure were then performed with categorical variables reaching significance in the univariate analysis. Different models were tested to compare the impact of PCT or sepsis score inclusions or not and areas under the ROC curves (AUC) were calculated both for the models and for each of the predictive variables, to compare if one model has one a better sensitivity/specificity than PCT or sepsis score alone. Optimal ROC curves were established with categorical variables, using a probability score to predict early sepsis diagnosis derived from a multivariate regression equation, as described by Shapiro *et al *[20]. The relationship between two parametric or non parametric variables was assessed using both InStat version 3.0 for basic between-group comparisons, SPSS version 16.0 (Chicago, IL, USA) for logistic regression analyses and MedCalc version 10 (Mariakerke, Belgium) for ROC calculations. *P *≤ 0.05 was considered statistically significant for all the performed tests.

## Results

### General patient data

General characteristics of the studied population and groups are detailed in Table 2. Given that it was an observational study, only the *post hoc *selected patients were included in the primary data analysis.

Healthy volunteers (n = 14) exhibited a similar profile to the ICU-admitted patients (age: 62.6 ± 3 and gender: 6/8) and their blood albumin content was 46.6 ± 0.5 g/L. Causes of MICU admission were: MI (n = 11); haemorrhage (n = 11), malignant arrhythmia (n = 3), ischemia (n = 3), drug overdose (n = 2), others (n = 7) for the non-sepsis group; the sites/origins of sepsis were: lung (n = 21), urinary tract (n = 7), soft tissues (n = 5), others (n = 4) for the sepsis group. Microbiological cultures in the sepsis group revealed infections by Gram positive/Gram negative bacteria (n = 16/17), multiple bacterial strains (n = 3) and fungi (n = 1). In-hospital mortality was 5/37 and 10/37 patients in the non-sepsis and sepsis groups, respectively (*P *= 0.247).

### Blood cortisol and ACTH determinations, corticotropin (Cortrosyn^®^) response and ACTH-to-cortisol ratio calculation

**Figure 2:**
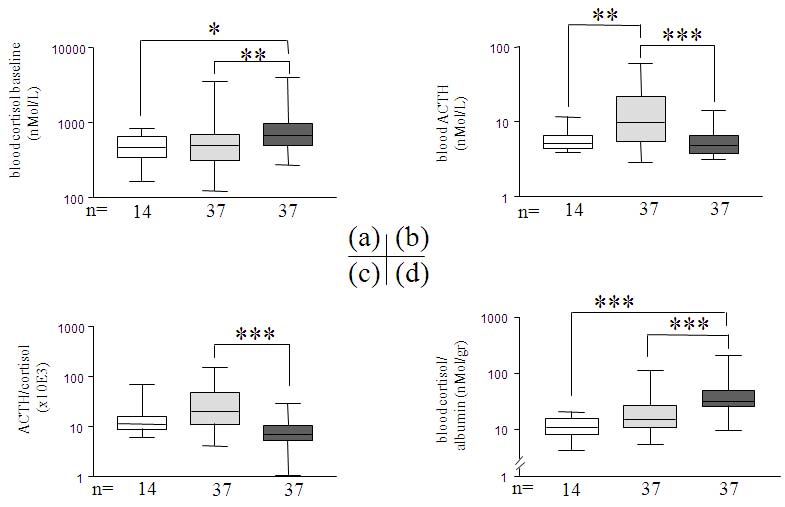
**On ICU-admission blood concentrations of cortisol; ACTH; ACTH-to-cortisol and cortisol-to-albumin ratios**. Three groups were compared: normal volunteers (white bars, n = 14), non-septic ICU patients (light grey bars, n = 37), septic ICU patients (dark grey bars, n = 37). Graphs represent analysis (median, box 25^th ^to 75^th ^percentile range; error bars 10th to 90^th ^percentile range) of **(a) **cortisol baseline (nMol/L), **(b) **ACTH (nMol/L), **(c) **ACTH-to-cortisol ratio, **(d) **cortisol-to-albumin. The Y axis is shown in logarithmic scale. *P *is indicative of significant difference(s) between groups: *: *P *≤ 0.05, **: *P *≤ 0.01, ***: *P *≤ 0.001.

High cortisol baseline on admission was associated with septic patients which displayed greater levels in comparison to non-septic patients and healthy volunteers (*P *< 0.05, Figure 2a). Correction for APACHE II scores still confirmed cortisol baseline as discriminant in early sepsis (unadjusted OR = 5.12; *P *= 0.005 vs. adjusted OR = 3.4; *P *= 0.048). However, septic patients and non-septic patients, as well as survivors/non-survivors, were not discriminated by excluding patients with evidence of CIRCI or by seeking the dynamic response of cortisol to corticotropin stimulation (NS). ACTH also displayed a differential profile with higher values in non-septic patients in comparison to septic patients and healthy volunteers (*P *< 0.01, Figure 2b). Consequently, an ACTH-to-cortisol ratio, as a marker of hypothalamo-pituitary-adrenal (HPA) corticotropic axis relationship [21], exemplified the dissociation between non-septic and septic values (*P *< 0.001, Figure 2c).

### Cortisol-to-albumin ratio calculation

The cortisol-to-albumin ratio as a marker of free-cortisol index [22] was selective with the highest values in septic vs. non-septic patients and healthy volunteers (*P *< 0.0001, Figure 2d).

### AVP, copeptin, APL, SDF-1α blood levels and ratios

**Figure 3:**
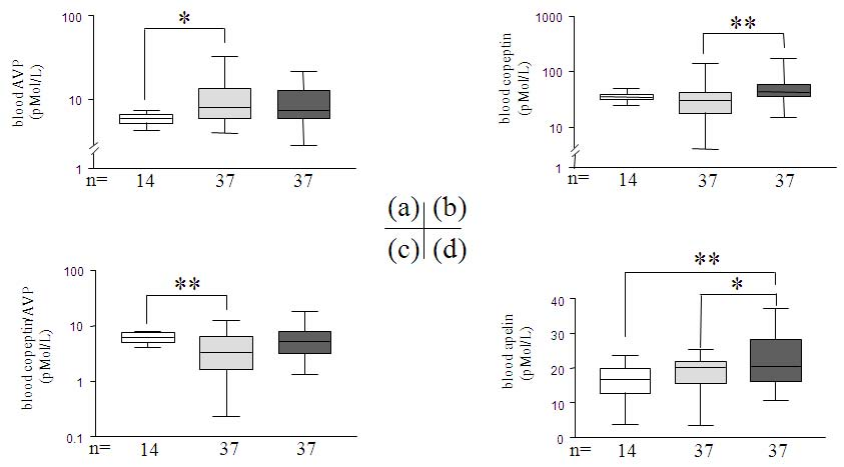
**On ICU-admission blood concentrations of arginine-vasopressin (AVP); copeptin; copeptin-to-AVP ratio, and apelin (APL)**. The three groups were compared in bar charts as described in Figure 1, and represent **(a) **AVP (pMol/L), **(b) **copeptin (pMol/L), **(c) **copeptin-to-AVP ratio, **(d) **apelin (APL, pMol/L). The Y axis is shown in logarithmic scale except for panel F which is linear. *P *is indicative of significant difference(s) between groups: *: *P *≤ 0.05, **: *P *≤ 0.01.

**Figure 4:**
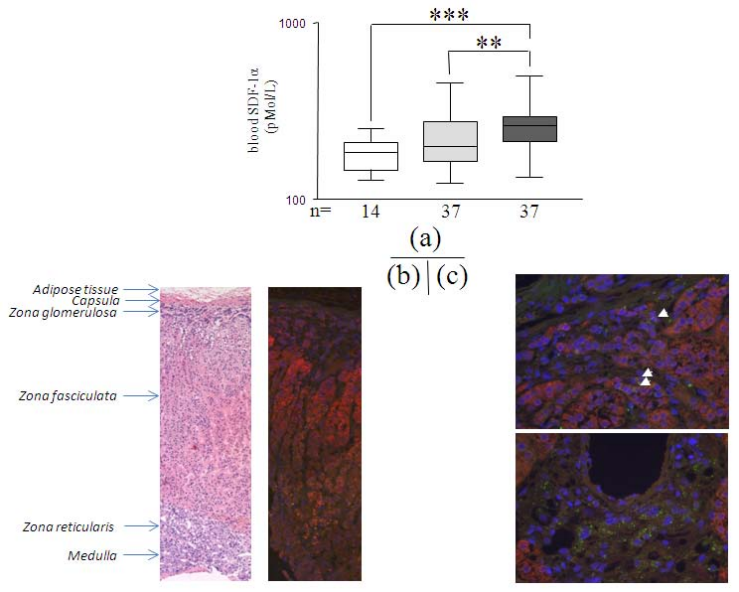
**On ICU-admission blood concentrations of SDF-1α(CXCL-12) and expression of its receptor (CXCR4) in adrenal gland**. The three groups were compared in bar charts as described in Figure 1, and represent **(a) **SDF-1α (pMol/L) in studied groups. The Y axis is in logarithmic scale. *P *are indicative of significant difference(s) between groups: **: *P *< 0.01, ***: *P *< 0.001. **(b) **low magnification of a human adrenal gland (× 40) stained with H&E; and after CP450-21-hydroxylase label (red), showing dominant specific expression in the zona fasciculata, **(c)** sparse expression of the SDF-1α receptor CXCR4 (green labeling, white arrows) by CP450-21-hydroxylase expressing cells of a human adrenal gland cortex (zona fasciculata, red labeling, magnification × 400) (upper panel), and dominant expression of CXCR4 (green labeling) by adrenal vascular wall cells, surrounded by CP450-21-hydroxylase expressing clusters in zona fasciculata (red labeling, magnification × 400) (lower panel).

Copeptin distinguished septic from non-septic patients better than AVP (*P *< 0.01, Figure 3a, b). Copeptin-to-AVP ratio, as an optimizing index, delineated decreased levels in non-septic MICU patients (*P *< 0.01 vs. healthy volunteers, Figure 3c). APL was slightly elevated in septic patients (*P *< 0.05 vs. both non-septic patients and healthy volunteers, Figure 3d). SDF-1α was increased in MICU patients with greater enhancement in septic patients compared to both non-septic patients and healthy volunteers (*P *< 0.01, Figure 4a). The CXCR4 (SDF-1α and MIF receptor) labeling pattern was scattered throughout P450-21hydroxylase-positive cells in the adrenocortical zona fasciculata. Intense punctiform staining was observed in regions surrounding adrenal vascular wall cells (Figure 4b, c).

### Circulating procalcitonin (PCT) content in critical illness

**Figure 5:**
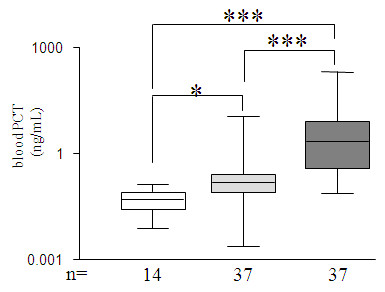
**On ICU-admission blood concentrations of procalcitonin (PCT)**. The three groups were compared in bar charts as described in Figure 1, representing PCT (ng/mL) in studied groups. The Y axis is in logarithmic scale. *P *are indicative of significant difference(s) between groups: *: *P *≤ 0.05, ***: *P *≤ 0.001.

Blood PCT was generally elevated in septic patients and remained distinctive from non-septic patients and healthy volunteers (*P *< 0.001, Figure 5). However, PCT was also greater in non-septic patients vs. healthy volunteers (*P *< 0.05, Figure 5).

### Correlations between SDF-1α/cortisol and AVP/cortisol

**Figure 6:**
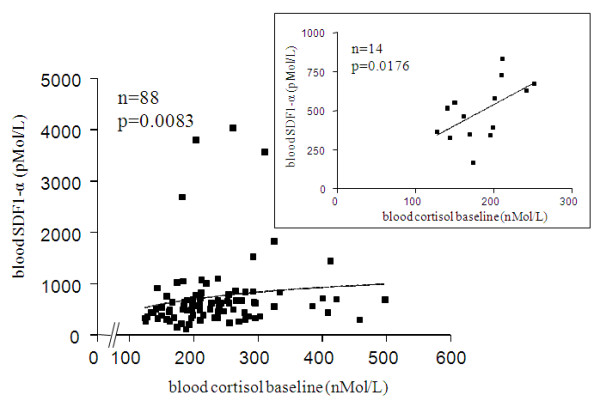
**Correlation between blood SDF-1α and cortisol baseline**. The overall studied population sample (including normal subjects) is shown included in the main panel: r = 0.2827 (95% CI: 0.06899 to 0.4717). The insert shows the strongest association between these two parameters found in healthy volunteers after subgroup analyses: r = 0.6220, (95% CI: -0.1191 to 0.8709).

**Figure 7:**
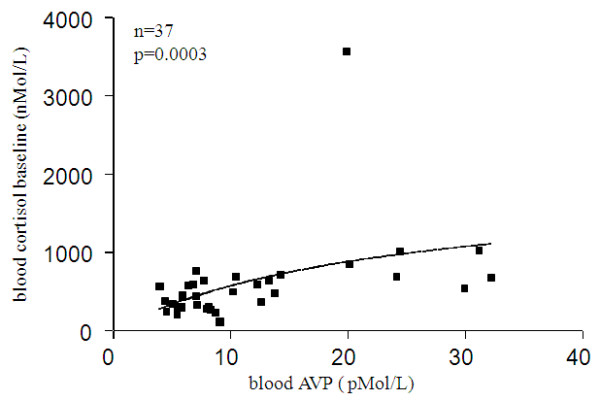
**Correlation between blood AVP and cortisol baseline**. The strongest association between these two parameters is shown in the non-septic ICU patient group: r = 0.5765 (95% CI: 0.2917 to 0.7674).

SDF-1α and AVP were positively correlated with their cortisol baseline counterpart level, in the overall study cohort (Figure 6), and in healthy volunteers after subgroup analysis (Figure 6 insert) for the former, and in the overall cohort and in ICU stress patients, with the strongest association observed in the nonseptic subset after subgroup analysis (Figure 7) for the later.

### Outcome value of neuro-corticotropic blood screening after MICU admission

Three neuro-corticotropic blood parameters were significant distinctive biomarkers among survivors (n = 59, 27 sepsis vs. 32 non-sepsis) and non in-hospital- survivors (n = 15, 10 sepsis vs. 5 non-sepsis): copeptin, cortisol baseline, and cortisol-to-albumin ratio (*P *= 0.045; *P *= 0.012; *P *= 0.0001, respectively).

### Prediction of sepsis diagnosis on ICU admission: usefulness of neuro-corticotropic blood screening in interpretative risk assessment and performance of a probability score

Individual receiver operating curve (ROC) factor analysis of general and molecular parameters allowed to delineate thresholds for further logistic regression studies, and the cutoff point corresponding to the best separation of data with minimal false negative and positive results was selected for studying categorical parameters. Univariate analysis identified six factors relative to sepsis diagnosis on ICU admission (Table 3). In order to screen whether the presence of at least two variables will perform better than a single recommended gold standard parameter (that is, sepsis score or PCT) to predict sepsis on admission, two new combinations of at least two out of three variables have been created. One of these was performant if at least two out of the three categorized variables were positive (that is .ACTH ≤ 233 nMol/L, cortisol baseline ≥ 450 nMol/L or PCT > 2 ng/mL), with an OR much higher than PCT alone (Table 3) and an AUC of 0.845 (0.738 to 0.920, 95% CI) vs. 0.726 (0.607 to 0.826, 95% CI) (*P *= 0.035). The other one (that is, ACTH ≤ 233 nMol/L, cortisol baseline ≥ 450 nMol/L or sepsis score >7) was also performant (Table 3) but AUCs were similar (0.811 (0.700 to 0.895, 95% CI) vs. 0.727 (0.607 to 0.827, 95% CI), *P *= 0.076).

**Table 3:** Prediction of sepsis diagnosis on admission: univariate regression analyse* (n = 74 patients)

	Odds Ratio (OR) (lower to upper 95% CI)	*P*-value
Cortisol baseline ≥ 450 nM/L	5.120 (1.623 to 16.156)	*0.004*
Apache II score ≥ 14	4.895 (1.652 to 14.503)	*0.003*
Shock	5.454 (1.841 to 16.159)	*0.001*
Sepsis score >7	6.48 (2.34 to 17.952)	*0.0002*
ACTH ≤ 233 nM/L	7.041 (2.446 to 20.271)	*0.0001*
Procalcitonin (PCT) >2 ng/ml	16.889 (3.523 to 80.959)	*0.0001*
At least two out of: cortisol, ACTH, sepsis score †	19.200 (5.741 to 64.210)	*< 0.0001*
At least two out of: cortisol, ACTH, PCT †	31.071 (8.215 to 117.516)	*< 0.0001*

ACTH, adreno corticotropic hormone; CI, confidence interval; OR, odds ratio; PCT, procalcitonin.

*: only significant factors are expressed (*P *< 0.05).

†: according to individual cut-offs values.

**Figure 8:**
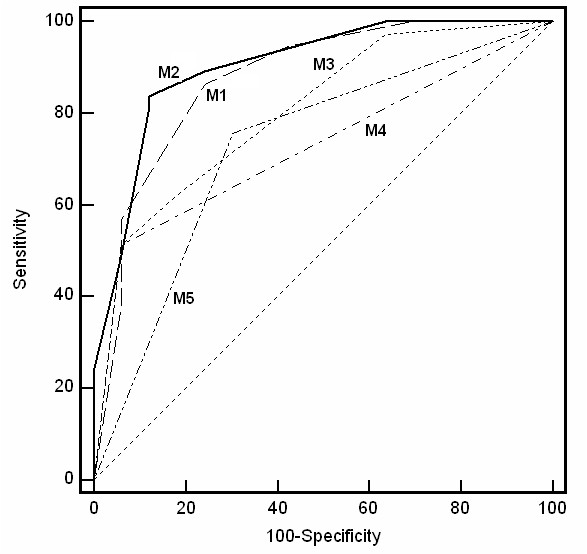
**Comparative Receiver operating characteristic curves for early sepsis diagnosis**. The curves relate the different models of logistic regression detailed in Table 4: **M1**: sepsis score >7; ACTH ≤ 233 nM/L; cortisol ≥ 450 nM/L, **M2**: PCT >2 ng/mL; ACTH ≤ 233 nM/L; cortisol ≥ 450 nM/L, **M3**: ACTH ≤ 233 nM/L; cortisol ≥ 450 nM/L, **M4**: PCT >2 ng/mL, **M5**: sepsis score >7. Sepsis score is a recommended clinico-biological multiple variable score [17,18], ACTH and cortisol baseline are a combination of two HPA biological stress parameters, and blood PCT is a gold standard biological marker of sepsis [13,50]. Respective AUC values are expressed in Table 4. The three combination panels including the two selected gold standards: sepsis score or PCT (in M1 and M2), performed for the best and similarly and offered added-value to each selected gold standard individually (that is, sepsis score (in M5) or PCT (in M4)): *P *= 0.005 and *P *< 0.001, respectively. On the other hand, adding sepsis score or PCT to the two combinations of stress parameters (ACTH, cortisol baseline) also optimized the prediction vs. ACTH and cortisol baseline: *P *= 0.037 and *P *= 0.036, respectively. Vertical axis represents the number of true positive values (sensitivity) and horizontal axis the number of false positive values (1-specificity), with the diagonal segments produced by ties.

Different multivariate logistic regression with stepwise method were performed to identify which of the significant variables in the univariate analyse perform better to assess sepsis diagnosis on admission (Table 4). ROC analysis was used to represent the probability score predicting early sepsis diagnosis from equations and to compare different models of regression. A first model was performed with categorized sepsis score, and PCT as well as shock were excluded because already part of its calculation. Another model was constructed by substituting sepsis score by PCT. These two models (M1 and 2) performed similarly (Table 4, *P *= 0.494) but were more efficient than the model (M3) including only categorized ACTH and cortisol baseline (*P *= 0.036 and = 0.0037, respectively). The best AUCs are shown in Figure 8.

**Table 4:** Different models of logistic regression to predict sepsis diagnosis on admission * (n = 74 patients)

	AUC (95% CI)	OR (95% CI)	*P*-value
**Model 1 (M1)**			
ACTH ≤ 233 nM/L	0.875 (0.774 to 0.942)	9.192 (2.466 to 34.265)	*0.001*
Cortisol baseline ≥ 450 nM/L		4.279 (1.007 to 18.181)	*0.049*
Sepsis score >7		5.371 (1.567 to 18.404)	*0.007*
**Model 2 (M2)**			
ACTH ≤ 233 nM/L	0.903 (0.808 to 0.960)	21.16 (4.252 to 105.307)	*0.0002*
Cortisol baseline ≥ 450 nM/L		8.81 (1.616 to 48.037)	*0.012*
Procalcitonin (PCT) >2 ng/mL		28.558 (4.393 to 185.636)	*0.0004*
**Model 3 (M3)**			
ACTH ≤ 233 nM/L	0.805 (0.693 to 0.890)	9.125 (2.667 to 31.218)	*0.0004*
Cortisol baseline ≥ 450 nM/L		7.457 (1.89 to 29.426)	*0.004*
**Model 4 (M4)**			
Procalcitonin (PCT) >2 ng/mL	0.726 (0.607 to 0.826)	16.889 (3.523 to 80.959)	*0.0004*
**Model 5 (M5)**			
Sepsis score >7	0.727 (0.607 to 0.827)	6.481 (2.34 to 17.952)	*0.0003*

ACTH, adreno corticotropic hormone; AUC, area under the receiver operating characteristic curve; CI, confidence interval; OR: odds ratio; PCT, procalcitonin.

## Discussion

The present study highlights differentially profiled stress responses consecutive to ICU admission between septic and non-septic patients. Septic patients exhibited higher cortisol baseline but decreased blood ACTH-to-cortisol ratio, and higher SDF-1α, copeptin, and apelin on admission. Copeptin, cortisol baseline and ACTH on admission were higher in septic non-survivors, while SDF-1α as well as AVP, in non-septic patients, was correlated with cortisol baseline. A probability score of biomarkers combining: baseline cortisol, ACTH, and PCT, was the best biological predictor of sepsis diagnosis and offers a substantial added value to each parameter individually or sepsis score.

### Corticotroph response to acute stress after ICU admission

Total cortisol baseline is clearly elevated in critical illness, but its outcome value is confusing [23-29]. In this study, higher cortisol baseline was observed in non-survivors, especially septic patients, as observed in large cohort studies [30,31]. The dynamic response of cortisol to corticotropin was not predictive of the outcome in our and other hands [23,27], in contradiction with the findings of Annane *et al *[5]. From these viewpoints, it appears imperative to thoroughly define study populations (that is, proper matching for period on inclusion, gender and age), sample timing, as well as to standardize methods of cortisol measurement, all being critical factors for comparisons.

Free cortisol index (FCI) has been advocated as a better determinant of the HPA status than cortisol in stressed patients [32], although not recommended for routine use by a recent international task force [4]. However, FCI requires cortisol binding globulin (CBG) measurement and total cortisol-to-albumin ratio can be an efficient and simplest surrogate marker [26,29,32-34]. In our study, total cortisol-to-albumin ratio was very high during acute sepsis and presented the best *on admission *outcome predictor.

### Differential dissociation of ACTH and cortisol in acute stress after ICU admission

ACTH-to-cortisol ratio dissociation, with low ACTH and high cortisol levels, is particularly observed during the chronic phase of critical illness [22], whereas high cortisol and ACTH have been observed during the initial phase [35,36]. Indeed, time-course and specificity of ACTH-to-cortisol dissociation are essential issues. In a small study [35], blood ACTH dramatically dropped in critically ill patients from Day 3 to 4, whereas cortisol remained high. An earlier dissociation was, however, observed on the first to second day in postoperative non-septic cancerous patients [37]. Furthermore, although ACTH-to-cortisol baseline ratio was reported decreased in critically ill non-survivors [23,25], it was either found unchanged in septic non-survivors [24] or lowered in septic patients [26]. In this study, we found a decrease in ACTH-to-cortisol baseline ratio distinctive of sepsis among critically ill patients, as a result of both blunted ACTH content and elevated cortisol. This suggests non-pituitary-driven sources and non-ACTH regulators of cortisol release during acute sepsis or HPA axis alteration inducing secondary adrenal dysfunction with sustained increased blood cortisol as a result of tissue resistance or impaired clearance [38]. Moreover, systemically or locally released neurotransmitters (catecholamines, AVP), neuropeptides, inflammatory cytokines (including IL-1, IL-6, MIF) and growth factors have been committed in this respect [38,39].

### Neuropeptides and the corticotropic HPA axis in acute stress after ICU admission

SDF-1α/CXCL12 colocalizes with AVP and APL in the magnocellular neurons of SON and PVN [12]. Centrally, SDF-1α can autoregulate AVP release through its receptor (CXCR4) [12,40]. Systemically, SDF-1α is released in bloodstream early after critical illness initiation, and is associated with endothelial progenitor cell mobilization, sepsis-induced organ dysfunctions [41,42]. Indeed, in this study, blood SDF-1α discriminated sepsis, as well as sepsis severity and outcome. A significant association was also found between blood SDF-1α and cortisol baseline, and clusters of CXCR4-expressing cells scattered were observed within the adrenal corticosteroid productive area. This suggests the existence of a peripheral cross-talk between SDF-1α, CXCR4 (also alternative receptor to CD74 for MIF [43]) and the HPA corticotroph axis. Apelin (APL) is another *counterpartner *peptide with centrally-driven regulatory activities on AVP and ACTH release, and peripherally-driven diuretic and non-ACTH-dependent cortisol release effects [9,11]. In this cohort of patients, blood APL was modestly elevated in critical illness, especially in sepsis, with no evidence of correlations found with either severity or outcome, nor with corticotroph HPA axis components.

Copeptin was also a more reliable diagnostic marker of sepsis than AVP in this study, as it was described as predictive of severity and outcome with more sustained blood levels than AVP in earlier works [12-16]. On the other hand, AVP was closely associated with cortisol baseline in our study, but only in non-septic ICU patients, further suggesting distinctive stress pathways in sepsis. Of note, higher blood AVP in septic shock has been observed with glucocorticoid administration [44] and a blunted cortisol response to corticotropin [45]. Also, a combination of glucocorticoids and AVP treatments was associated with improved survival and increased vasopressor-free days [44,46,47], as well as with reversal of AVP hyporesponsiveness in sheep [47]. This suggests a possible *reset *of sepsis-induced vascular V1aR down-regulation through the GRE receptor gene [48,49], and resurfaces a complex and often questioned link between AVP and corticosteroids, which is essentially disturbed in critical illness.

### A neurocorticotropic marker combination for sepsis diagnosis in acute stress after ICU admission

Procalcitonin (PCT) is a known biochemical reference marker of sepsis diagnosis and severity [13,50,51], and sepsis score is a tentative summation of clinical and biochemical variables (including PCT) [18]. However, using the cutoff recommended by the manufacturer, PCT was not a perfect biomarker, as well as sepsis score, on diagnostic prediction. Conversely, a combination of the two categorized variables (cortisol baseline ≥ 450 nMol/L, ACTH ≤ 233 nMol/L) performed equally to PCT (>2 ng/mL), and a predicted probability score for sepsis diagnosis using these all three categorical bio-variables (that is, categorized PCT or sepsis score) substantially improved the initial prediction of sepsis diagnosis in this cohort.

### Limitations of the study

This study is observational and somewhat limited because of the nominal number of included patients, although thoroughly matched with regard to timing inclusion and general characteristics of the study population. Although our initial *gold standard diagnosis *for sepsis was a clinical one with a relatively low likelihood ratio, it was further validated by a microbiological confirmation, but re-allocations were mandatory. Selection of parameters, especially neurocorticotropic markers, while arbitrary, was nonetheless based on recent knowledge relative to coexpression of studied neuropeptides and cytokines (SDF-1α, AVP, copeptin, APL) in the CNS. A not-highly sensitive PCT measurement has been selected. In the multiple regression analysis, the use of more than three variables with a limited sample did not definitely avoid possible overfitting. The alternative diagnostic combination of parameters (cortisol baseline, ACTH) proposed to challenge PCT measurement in early sepsis diagnosis is not necessarily always easier or faster to obtain in all centers. Of course, a larger study should further validate the diagnostic usefulness of this biomarker combination.

## Conclusions

The neuro-corticotropic systemic stress response of early admitted ICU patients is differentially profiled with special emphasis on sepsis. An alternative diagnostic combination of categorical parameters (cortisol baseline, ACTH) was as efficient as PCT or sepsis score in identifying critical sepsis, and all together offered the best performance. This might help ICU physicians in refining bedside diagnostic tools, in addition to traditional microbiological sampling and decision-making strategies, and calls for further validation on a larger population.

## Key messages

• The neuro-corticotropic stress response (ACTH/cortisol baseline, copeptin, apelin, SDF-1α) is in someway different in septic vs. non-septic early admitted ICU patients.

• Adding cortisol baseline and ACTH to PCT blood measurement or sepsis score -gold standards- in a combination of variable score analyses helps in refining bedside diagnostic tools to efficiently diagnose sepsis.

## Abbreviations

ACTH: adreno corticotropic hormone; APL: apelin; AUC: area under the curve; AVP: arginine vasopressin; CBG: cortisol binding globulin; CIRCI: critical illness-related corticosteroid insufficiency; CNS: central nervous system; CORTICUS: Corticosteroid therapy of septic shock study; CRH: corticotrophin-releasing hormone; CXCR4: C-X-C chemokine receptor type 4; FCI: free cortisol index; GRE: glucocorticoid response element; HPA: hypothalamo pituitary adrenal; MIF: Macrophage migration Inhibitory Factor; (M)ICU: (Medical) Intensive Care Unit; MI: myocardial infarct; PCT: procalcitonin; PVN: paraventricular nuclei; ROC: receiver operating characteristic; SDF-1α: stromal-derived factor-1 alpha; SIRS: systemic inflammatory response syndrome; SON: supraoptic.

## Competing interests

The authors declare that they have no competing interests.

## Authors' contributions

OL, JFR, FC, NG-P, RD, PS and AC participated in the study design. OL, JFR, FC, LC and BH performed the study. OL, JFR, FC and LC processed the data and performed the statistical analysis. OL and FC wrote the manuscript. All authors read and approved the final manuscript.
